# The Impact of Digital Patient Portals on Health Outcomes, System Efficiency, and Patient Attitudes: Updated Systematic Literature Review

**DOI:** 10.2196/26189

**Published:** 2021-09-08

**Authors:** Elettra Carini, Leonardo Villani, Angelo Maria Pezzullo, Andrea Gentili, Andrea Barbara, Walter Ricciardi, Stefania Boccia

**Affiliations:** 1 Section of Hygiene University Department of Life Sciences and Public Health Università Cattolica del Sacro Cuore Rome Italy; 2 Hygiene and Public Health Service ASL Roma 1 Rome Italy; 3 Department of Public Health and Infectious Diseases Sapienza University of Rome Rome Italy; 4 Department of Women, Children and Public Health Sciences - Public Health Area Fondazione Policlinico Universitario A Gemelli IRCCS Rome Italy

**Keywords:** digital health, patient portal, health outcomes, patient satisfaction, patient attitudes, health service research, health care efficiency

## Abstract

**Background:**

Patient portals are becoming increasingly popular worldwide even though their impact on individual health and health system efficiency is still unclear.

**Objective:**

The aim of this systematic review was to summarize evidence on the impact of patient portals on health outcomes and health care efficiency, and to examine user characteristics, attitudes, and satisfaction.

**Methods:**

We searched the PubMed and Web of Science databases for articles published from January 1, 2013, to October 31, 2019. Eligible studies were primary studies reporting on the impact of patient portal adoption in relation to health outcomes, health care efficiency, and patient attitudes and satisfaction. We excluded studies where portals were not accessible for patients and pilot studies, with the exception of articles evaluating patient attitudes.

**Results:**

Overall, 3456 records were screened, and 47 articles were included. Among them, 11 studies addressed health outcomes reporting positive results, such as better monitoring of health status, improved patient-doctor interaction, and improved quality of care. Fifteen studies evaluated the impact of digital patient portals on the utilization of health services with mixed results. Patient characteristics were described in 32 studies, and it was reported that the utilization rate usually increases with age and female gender. Finally, 30 studies described attitudes and defined the main barriers (concerns about privacy and data security, and lack of time) and facilitators (access to clinical data and laboratory results) to the use of a portal.

**Conclusions:**

Evidence regarding health outcomes is generally favorable, and patient portals have the potential to enhance the doctor-patient relationship, improve health status awareness, and increase adherence to therapy. It is still unclear whether the use of patient portals improves health service utilization and efficiency.

## Introduction

In recent years, electronic tools that allow patients to interact with health care professionals have considerably increased with consequences on the awareness of citizens about their own health [1]. The adoption of these technologies might represent an important measure to improve the quality and efficiency of health care services and is a key feature for the construction of a more equitable, effective, and safe health care system [2]. Indeed, the rapid growth and diffusion of digital health, including health information sources, such as electronic medical records (EMRs), has made online access to information by patients and health care professionals a crucial component of health care delivery [3].

In this context, patient portals are thought to allow patients secure access to health-related information and to communicate and share information with providers [4]. Besides guaranteeing protected access to EMRs, more advanced patient portals allow secure message exchange between health professionals and citizens, consultation of educational material adapted to patients’ own characteristics, appointment scheduling, automatic renewal of medical prescriptions for chronic diseases, and facilitation of payments. Despite their potential benefits, several studies have proved underuse or inappropriate use of patient portals and their limited impact [5]. Furthermore, the majority of studies available on this topic have focused on users’ characteristics and satisfaction, and few studies have considered the consequences on health outcomes [6-8]. Patient portals are relatively new technologies with continuous updates. Several types are released every year, and this may explain the lack of research in this area [5].

A systematic literature review in 2013, which addressed the effect of patient portals on patient clinical care, reported that evidence was limited to evaluate whether patient portals had a positive, negative, or neutral impact on users’ health [4]. Some of the most effective examples refer to patients with chronic diseases, such as diabetes, hypertension, and depression, specifically when the portal is included in a shared health care pathway [9-13]. The effect of patient portals on health care utilization and efficiency, instead, is unclear due to the scarcity of studies examining the impact of patient portals on key indicators, such as inpatient hospitalizations, emergency department (ED) and outpatient visits, length of stay, and telephone contacts [14]. The aim of this systematic review was to update the study performed in 2013, by summarizing evidence on the impact of digital patient portals on patients’ health outcomes, health care efficiency, and patients’ attitudes and satisfaction.

## Methods

### Search Strategy

A search of relevant articles was performed in the PubMed and Web of Science databases using the query reported in Multimedia Appendix 1. The resulting records were entered in a dedicated work sheet to be subsequently screened according to the inclusion/exclusion criteria. Following the removal of duplicates, the selection was made by reading titles and abstracts, and then the full texts.

### Inclusion/Exclusion Criteria

Eligible studies were primary studies reporting on the impact of patient portal adoption in relation with health outcomes, health care efficiency, and patients’ attitudes and satisfaction. Articles included were published from January 1, 2013, to October 31, 2019, and written in English, Italian, Spanish, or French. We excluded studies describing portals that were not accessible for patients, as well as pilot studies, with the exception of studies evaluating patients’ attitudes.

### Selection Process and Data Extraction

Two authors screened the articles, and each reference retrieved was screened by two researchers independently, with any disagreement finally discussed and resolved by a third researcher, if necessary. The following information was extracted from the studies: first author name, publication year, study country, study design, study population, study setting, study duration and time, health information technology, study objective, main findings according to health outcomes, health care efficiency/utilization, patient characteristics, and patient attitudes and satisfaction. The systematic literature review was conducted according to the PRISMA (Preferred Reporting Items for Systematic Reviews and Meta-Analyses) 2009 checklist [15].

## Results

### Characteristics of the Included Studies

**Figure 1:**
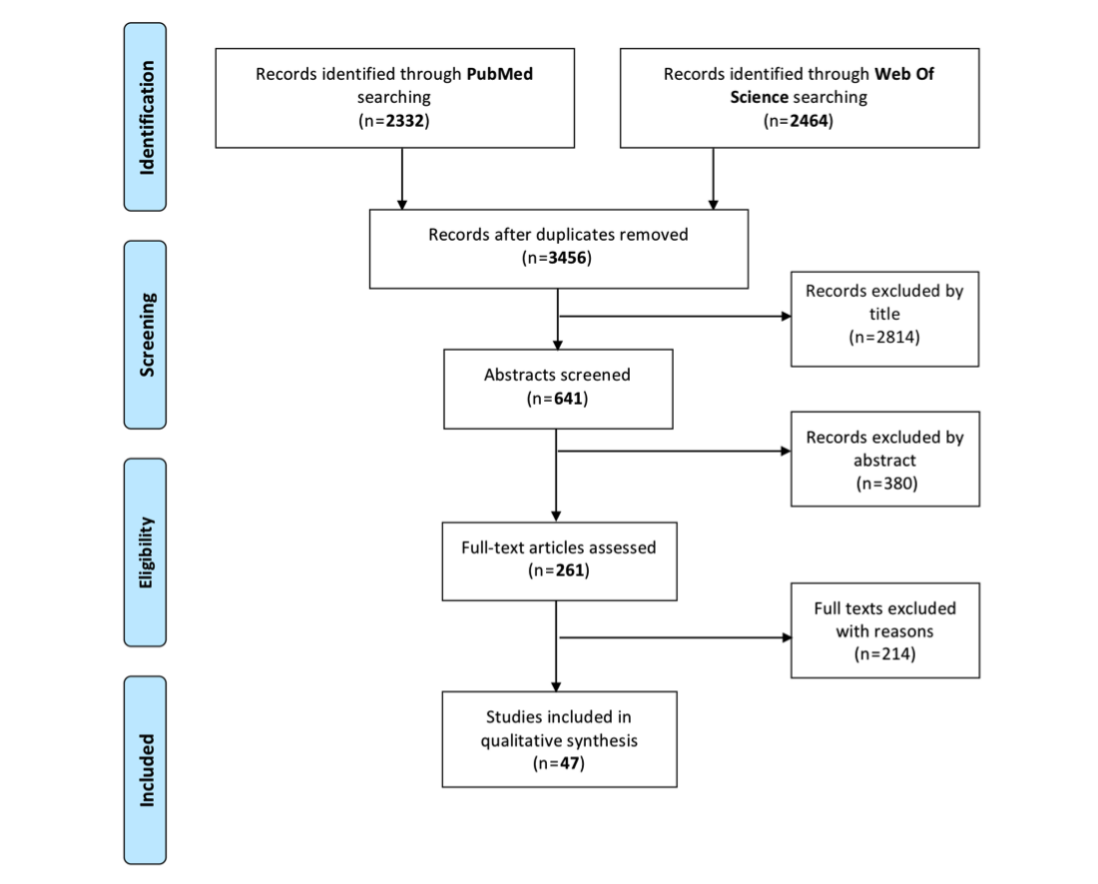
PRISMA (Preferred Reporting Items for Systematic Reviews and Meta-Analyses) flow diagram.

The database search, after duplicate removal, identified a total of 3456 records. According to the inclusion/exclusion criteria, the screening resulted in the inclusion of 47 full-text articles (Figure 1).

The study designs were grouped into six categories according to the characteristics of the articles. Overall, 17 were descriptive quantitative studies [8,16-31], two were descriptive mixed-methods studies [32,33], 14 were observational hypothesis testing studies [20,34-46], seven were descriptive qualitative studies [47-53], five were interventional studies, other than randomized controlled trials (RCTs) [54-58], and three were RCTs [59-61].

With regard to country, 33 (70%) studies were based in the United States [17,18,20,21,31,32,34,43-46,49,60], three in Canada [35,36,50], three in the Netherlands [19,26,57], two in Finland [55,56], two in the United Kingdom [16,48], one in Australia [22], one in France [59], one in Israel [47], and one in Sweden [24].

Various patient portals have been described in the studies. Multimedia Appendix 2 provides details on the functionalities of the portals, and Multimedia Appendix 3 provides qualitative descriptions of the portals.

Most of the portals were not addressed to a defined population subgroup, and only some of them were specific to a clinical specialty/condition, such as endocrinology-diabetes [8,21,26,34,36,41], primary care [33,37,38,40,60], mental health [31,35,54], multiple chronic conditions [40,55,56], pulmonology and asthma [32,44,60], rheumatology [50,57,59], cardiology [37,44], internal medicine [40,61], nephrology [30,46], pregnancy [34], cancer [53], and gastroenterology [48].

The population included in the studies was heterogeneous in terms of sample size (from 24 [52] to 2,171,325 patients [31]) and groups of included patients (eg, pediatric [32], older [31], oncology [52], and diabetic patients [60]).

The results were summarized in four categories, albeit the same study could belong to more than one category. In particular, 11 studies analyzed health outcomes and adherence, intended as a change in individual or population health, attributable to health-related interventions. Adherence is the degree to which a patient follows medical advice, especially drug compliance. Overall, 15 studies focused on health care efficiency/utilization (utilization of health care services), 32 studies referred to patient characteristics, and 30 studies analyzed attitudes and satisfaction.

### Health Outcomes and Adherence

Eleven articles presented results on patient outcomes (Table 1), such as prevention, diabetes, blood pressure control, asthma, mental health, and medication adherence.

In particular, a significant association was found between patients’ preventive health behaviors and portal use [45]. Considering diabetes [34,36] and blood pressure control [37], portal users were significantly more likely to control their HbA_1c_ levels successfully compared to nonusers.

An improved clinical condition over time was observed in the management of asthma in children, where the group using the patient portal reported better flare control compared to the control group [60].

Concerning mental health, patients using the portal had a positive impact related to the clinical condition in many domains [35], such as the reduction of drinking days [54]. However, concerning the health status, no marked short-term impact was described, and differences in patient-reported physical and mental health changes were minor [55].

Finally, higher medication adherence was described in portal users compared with nonusers, especially among pediatric patients with asthma and patients with rheumatic disorders [32,57].

**Table 1:** Summary of the findings on health outcomes and adherence.

First author, year	Country	Health information technology	Study design	Sample size	Main findings
Ancker, 2019 [34]	United States	Blood glucose flow sheet (EpicCare and Weill Cornell Connect portal)	Observational, hypothesis testing	53 patients	Pregnant: average BMI dropped while average blood pressure increased significantly more in the 9 months among uploaders than nonuploaders. Chronic disease patients: after 9 months, uploaders had significantly larger reductions in HbA_1c_ and BMI than nonuploaders. One subset of uploaders had low well-controlled HbA_1c_ values before and during PGHD^a^ upload. Another uploader subset began to upload when their HbA_1c_ levels were elevated and experienced a decrease in HbA_1c_ levels followed by a plateau.
Fiks, 2015 [60]	United States	MyAsthma (clinical interface in MyChart)	RCT^b^	60 families of children	No significant differences in baseline control, quality of life, or parent activation between the two study arms (*P*>.2 for all comparisons). Frequency of asthma flares improved in the intervention group over time by 2.0 points on a 25-point scale (*P*=.02). Families in the intervention group had a marginally significant improvement in symptoms during periods without flares. A nonstatistically significant improvement in quality of life in terms of daytime symptoms and functional limitations was observed in the intervention group. There were no significant changes in parent activation.
Fiks, 2016 [32]	United States	MyAsthma	Descriptive, mixed methods	237 families	Portal users with uncontrolled asthma had significantly more medication changes after using the portal relative to the year earlier (increase of 14%).
Huang, 2019 [45]	United States	MyPennMedicine (branded version of Epic MyChart)	Observational, hypothesis testing	10,000 patients	Patients’ preventive health behaviors were significantly associated with portal use. The proportions of annual flu vaccination, blood pressure checks, and lipid level screening were substantially higher in portal users compared with nonusers (OR^c^=1.58, 1.13, and 1.50, respectively; *P*<.001). The average composite prevention score was significantly higher among portal users compared with nonusers (mean difference=0.22; *P*<.001). The proportion of colorectal cancer screening between users and nonusers was statistically significant (*P*<.001, OR very close to 1). No clinically meaningful difference between patient portal users and nonusers in chronic health outcomes.
Jhamb, 2015 [46]	United States	Free patient portal tethered to an ambulatory EHR^d^	Observational, hypothesis testing	1098 patients	In the fully adjusted model (controlling for hyperlipidemia, nephrolithiasis, history of kidney transplant, CCI^e^, proteinuria, eGFR^f^, number of nephrology and outpatient visits, and university affiliated PCP^g^), the association was not significant (OR 1.11, 95% CI 0.99-1.24).
Kipping, 2016 [35]	Canada	Ontario Shores HealthCheck Patient Portal	Observational, hypothesis testing	91 patients	The overall Mental Health Recovery Measure score increased from 70.4 (SD 23.6) at baseline to 81.7 (SD 25.1) at follow-up (*P*=.01). Of the eight domains, seven increased from baseline to follow-up (overcoming stuckness, self-empowerment, basic functioning, overall well-being, new potentials, spirituality, and advocacy/enrichment; all *P*<.05. No change for learning and self-redefinition).
Lau, 2014 [36]	Canada	BCDiabetes.ca	Observational, hypothesis testing	1957 patients	Overall, 28 of 50 users had a follow-up HbA_1c_ ≤7%, whereas 22 of 50 did not (56% success rate). Only 16 of 50 nonusers achieved a follow-up HbA_1c_ ≤7%, while 34 of 50 did not (32% success rate). Users were significantly more likely to control their HbA_1c_ levels successfully than nonusers (McNemar test, *P*=.03). The HbA_1c_ level at the last follow-up was significantly lower for users compared to nonusers (*P*=.02).
Manard, 2016 [37]	United States	Online patient portal	Observational, hypothesis testing	1571 patients	After adjusting for age, users were more likely to achieve BP^h^ control (HR^i^ 1.24, 95% CI 1.06-1.45). After adjustment for sociodemographics, portal use was no longer associated with BP control (HR 0.98, 95% CI 0.83-1.16).
Quanbeck, 2018 [54]	United States	Seva	Interventional, other than RCT	268 patients	Significant reductions in the numbers of risky drinking days, which declined by 44% ([0.7-1.25]/1.25) from baseline to 6 months, and illicit drug-use days, which declined by 34% ([2.14-3.22]/3.22). Two of the three abstinence outcomes showed significant improvements (any illicit drug use and/or any drink or drug). Significant effects were found for two of the three quality of life scores (overall quality of life and mental health).
Riippa, 2015 [55]	Finland	Patient portal by The Finnish Medical Society, Duodecim	Interventional, other than RCT	876 patients	Minor differences in patient-reported physical and mental health changes that changed the sign from the matched (physical health mean=1.2, 95% CI −3.3 to 5.7; mental health mean=0.8, 95% CI −3.6 to 5.2) to the unmatched sample (physical health mean=−0.4, 95% CI −4.7 to 3.9; mental health mean=−0.4, 95% CI −4.8 to 4.0). Patient activation improved more in the intervention group, but it was not statistically significant. There was no marked short-term impact on health status based on the SF-36v2 measure.
Van der Vaart, 2014 [57]	Netherlands	Medisch Spectrum Twente	Interventional, other than RCT	360 patients	Overall, 56% of the respondents had a score of 7 (out of 8) on medication adherence.

^a^PGHD: patient-generated health data.

^b^RCT: randomized controlled trial.

^c^OR: odds ratio.

^d^EHR: electronic health record.

^e^CCI: Charlson Comorbidity Index.

^f^eGFR: estimated glomerular filtration rate.

^g^PCP: primary care practice.

^h^BP: blood pressure.

^i^HR: hazard ratio.

### Efficiency/Utilization

Fifteen articles described the relationship between portal use and health care service efficiency and utilization (Table 2). The use of a digital portal had an effect on the utilization of health care services in terms of the number of clinical visits, especially for asthmatic patients [32,60], while no statistically significant changes in the number of primary care visits was reported in association with the use of secure messaging [38].

**Table 2:** Summary of the findings on health care efficiency.

First author, year	Country	Health information technology	Study design	Sample size	Main findings
Ancker, 2019 [34]	United States	Blood glucose flow sheet (EpicCare and Weill Cornell Connect portal)	Observational, hypothesis testing	53 patients	Uploaders had more clinical visits and portal logins before initial data upload.
Bidmead, 2016 [48]	England (United Kingdom)	Patients Know Best (PKB)	Descriptive, qualitative	56 patients	The portal enabled clinicians to manage stable patients, facilitating clinical and cost-effective use of specialist nurses, and improved two-way communication and more optimal use of outpatient appointments and consultant time. It also facilitated a single rationalized pathway for stable patients, enabling access to information and proactive support.
Fiks, 2015 [60]	United States	MyAsthma (clinical interface in MyChart)	RCT^a^	60 families of children	The intervention group had a marginally significant reduction in the proportion of parents missing at least 1 day of work (reduction of 47%, *P*=.07). Families in the intervention group reported fewer ED^b^ visits and hospitalizations for asthma over 6 months than the control group (3 vs 9 and 0 vs 2, respectively). Only two intervention families reported at least one ED visit (vs six control families), and no intervention families reported hospitalizations. Children in the intervention group had fewer visits with asthma specialists or primary care. Results were similar on stratifying by asthma severity.
Fiks, 2016 [32]	United States	MyAsthma	Descriptive, mixed methods	237 families	Portal users with uncontrolled asthma had significantly more primary care asthma visits after using the portal than the year earlier (increases of 16%).
Foster, 2019 [43]	United States	Epic MyChart	Observational, hypothesis testing	208,635 tests	ED visits: 80.56% (n=20,430) of patients had a single ED visit with laboratory testing, 16.04% (n=4069) had two or three ED visits, 3.16% (n=802) had four to 10 ED visits, and only 0.24% (n=60) had more than 10 ED visits. Activation rates were lower for those with only a single ED visit (7312/20,430, 35.79%) compared with either those with two to three ED visits (1770/4069, 43.50%; *P*<.001) or four or more ED visits (368/862, 42.7%; *P*<.001).
Griffin, 2016 [44]	United States	My UNC Chart	Observational, hypothesis testing	2975 patients	The odds of being readmitted within 30 days for active users was 66% higher than that for nonusers, holding all other variables constant in the model. There was no significant difference in 30-day readmission between nonusers and light users.
Jahn, 2018 [49]	United States	My HealtheVet	Descriptive, qualitative	29 participants	Secure messaging tasks were inefficient as related to clinical document sharing (it took almost 5 minutes for providers to only attach and send a clinical document).
Kipping, 2016 [35]	Canada	Ontario Shores HealthCheck Patient Portal	Observational, hypothesis testing	91 patients	Fewer missed appointments and a reduced number of requests for information in the year following portal implementation. The odds of a portal user attending an appointment were 67% (CI 56%-79%) greater than for nonusers over the follow-up period. Compared with 2014, in 2015, there was an 86% and 57% decrease in requests for information among users and nonusers, respectively (61% overall).
North, 2014 [38]	United States	Mayo Clinic Health System	Observational, hypothesis testing	2357 primary care patients	Primary care patients who sent at least one secure message or e-visit had a mean of 2.43 (SD 2.3) annual face-to-face visits before the first message and 2.47 (SD 2.8) after, with a nonsignificant difference (*P*=.45). After adjustment for a first message surge in visits, no significant visit frequency differences were observed (mean, 2.35 annual visits per patient both before and after the first message; *P*=.93). Subgroup analysis showed no significant change in visit frequency for patients with higher message utilization or for those who had used the messaging feature longer.
Plate, 2019 [39]	United States	MyChart; Epic Systems Corporation	Observational, hypothesis testing	6426 patients	Active MyChart status was not associated with 90-day ED return (*P*=.78) or readmission (*P*=.51) based on univariable analysis. Similarly, during multivariable analysis controlling for age, gender, BMI, and ASA^c^ category, active MyChart utilization was not significantly associated with 90-day ED visits (OR^d^ 1.019, 95% CI 0.843-1.231; *P*=.85) or readmissions (OR 0.966, 95% CI 0.747-1.249; *P*=.79). Patients who sent secure messages within 90 days from surgery (2200 patients, 48% of active users) were not less likely to present to the ED (*P*=.63) or be readmitted (*P*=.59) within 90 days. For patients who sent two or more messages (1354 patients), provider or staff response rate <75% was significantly associated with 90-day readmission (*P*=.004) with greater 90-day ED visits that neared statistical significance (*P*=.07).
Quanbeck, 2018 [54]	United States	Seva	Interventional, other than RCT	268 patients	Significant reduction in hospitalizations and a trend toward fewer ER^e^ visits. Increase in HIV screening rates. Change in the rates of HIV risk behaviors (eg, condom use) and receiving other addiction treatments appeared to be nonsignificant.
Riippa, 2015 [55]	Finland	Patient portal by The Finnish Medical Society, Duodecim	Interventional, other than RCT	876 patients	The effect on the cost of care was ambiguous; costs decreased by an average of €91 in the unadjusted model, but increased by €48 in the adjusted model. Due to the controversial result, the unadjusted analysis showed an 89% probability of cost-effectiveness with no willingness to pay for increased patient activation, whereas in the adjusted sample, the probability of the portal being more cost-effective than care as usual exceeded 50% at a willingness to pay €700 per clinically significant increase in the patient activation score. For doctor visits, portal access (n=80): 3.8 (SD 3.3) and control (n=57): 3.0 (SD 3.1) (*t*=1.4; *P*=.18). For nurse visits, portal access (n=80): 3.5 (SD 2.6) and control (n=57): 4.1 (SD 2.5) (*t*=−1.3; *P*=.18).
Tsai, 2019 [28]	United States	Epic’s personal health record system	Descriptive, quantitative	109,200 patients	Active users had more outpatient and inpatient visits and fewer ER visits. Patients without a portal account had on average fewer outpatient visits per month (0.31 vs 0.89, *P*<.001) and fewer inpatient visits per month (0.007 vs 0.059, *P*<.001), but had more ER visits per month than patients who were active with the portal (0.047 vs 0.014, *P*<.001). The difference between no-show appointments was not significant.
Wallace, 2016 [58]	United States	MyChart by Epic health record system	Interventional, other than RCT	36,549 patients	The number of visits for 12 months was strongly associated with an increased likelihood of MyChart activation and with more frequent MyChart logins.
Zhong, 2018 [42]	United States	MyUFHealth (also known as MyChart by Epic)	Observational, hypothesis testing	15,659 nonusers and 5494 users	At the time of adoption, the quarterly PCP^f^ office visit RR^g^ of users to nonusers was 1.33 (95% CI 1.27-1.39; *P*<.001). The RRs were between 0.94 and 0.99 up to four quarters after portal adoption (*P*=.75, .10, .13, and .09, respectively), and it was significantly less than 1 at the seventh (RR 0.82, 95% CI 0.73-0.91; *P*<.001) and eighth (RR 0.80, 95% CI 0.70-0.90; *P*<.001) quarters post adoption. The no-show rate proxies in the user group were significantly lower than in the nonuser group. RRs were between 0.60 and 0.83 for eight out of 11 quarters, and for the remaining three quarters, differences were not significant (*P*=.65, .29, and .44, respectively). Differences in cancellation rate proxies were not significant (*P*>.05). Overall, appointment adherence improved after portal adoption.

^a^RCT: randomized controlled trial.

^b^ED: emergency department.

^c^ASA: American Society of Anesthesiology.

^d^OR: odds ratio.

^e^ER: emergency room.

^f^PCP: primary care physician.

^g^RR: rate ratio.

Concerning emergency room (ER) visits, a decreasing trend was described [54,60], and active portal users showed more outpatient and inpatient visits and fewer ER visits per month, compared with patients without an account [28]. The number of visits over 6 months for asthmatic patients was lower among users than among nonusers (3 vs 9) [60]. User status was not significantly associated with ED access in the study by Plate et al, and the use of secure messages showed a nonsignificant result [39].

A reduction in hospitalizations was described for asthma [60] and mental health conditions [54]. Different findings were observed in patients with acute myocardial infarction, congestive heart failure, or pneumonia. In these cases, the odds of 30-day readmission for active users was 66% higher than that for nonusers, while no significant difference was described between nonusers and light users [44]. Considering patients who underwent total hip or knee arthroplasty, there was a significant association between 90-day readmission and sending two or more secure messages [39].

Concerning clinicians’ attitudes, the portal seemed to enable a new way of managing stable patients, facilitating clinical and cost-effective use of specialist nurses (improved two-way communication, and more optimal use of outpatient appointments and consultant time). The portal also facilitated a single rationalized pathway for stable patients, enabling access to information and proactive support [48].

Portal use by patients reduced missing appointments [35] and showed an improvement in appointment adherence after portal adoption [42]. However, no significant association between user/nonuser status and no-show appointments was found by one study [28]. The difference in cancellation rate proxies was also not significant between the user and nonuser groups [42].

Finally, concerning other aspects, only one study took into consideration the associated costs with ambiguous results. In this study, costs decreased in the unadjusted model but increased after adjusting for relevant variables [55]. An increase in HIV screening rates was described, but changes in the rates of HIV risk behaviors (eg, condom use) and modification of addiction treatments appeared nonsignificant in mental health patients [54]. Portal use had a positive effect on days of work lost due to asthma patients’ issues [60]. Moreover, information services were positively affected by portal use, as it led to a reduced number of requests [35].

### Patient Characteristics

Concerning patients’ characteristics, 32 articles presented relevant results that were mainly related to demographic information, such as age, gender, education, and household status (Table 3).

**Table 3:** Summary of the findings on patient characteristics.

First author, year	Country	Health information technology	Study design	Sample size	Main findings
Abel, 2018 [31]	United States	My HealtheVet (MHV) and Clinical Video Telehealth (CVT)	Descriptive, quantitative	2,171,325 patients	African American and Latino patients were significantly less likely to engage in use compared with White patients. Low-income patients with free care were significantly less likely to be users. Patients with schizophrenia or schizoaffective disorder were significantly less likely to be users than those with other mental health diagnoses (OR^a^ 0.50, CI 0.47-0.53 and OR 0.75, CI 0.69-0.80, respectively). Although rural patients had 17% lower odds of My HealtheVet adoption compared with urban patients (OR 0.83, 95% CI 0.80-0.87), they were more likely to engage in Clinical Video Telehealth and dual use (OR 2.45, 95% CI 1.95-3.09 for Clinical Video Telehealth and OR 2.11, 95% CI 1.81-2.47 for dual use).
Ancker, 2019 [34]	United States	Blood glucose flow sheet (EpicCare and Weill Cornell Connect portal)	Observational, hypothesis testing	53 patients	Pregnant patients: Uploaders were similar to the comparison group in terms of race, ethnicity, age, and socioeconomic status. Uploaders had more clinical encounters and portal logins before initial data upload, earlier establishment of patient portal accounts, and worse baseline blood pressure. 30 chronic disease patients: Uploaders were more likely to be Asian-American and younger, but the groups did not have other significant demographic differences.
Chan, 2018 [18]	United States	MyChart (EpicCare, Verona, WI)	Descriptive, quantitative	17,699 patients	Positive associations between portal use and being aged 50-74 years, White, privately insured, English-speaking, and living outside San Francisco.
Fiks, 2015 [60]	United States	MyAsthma (Clinical interface in MyChart)	RCT^b^	60 families of children	Parents of children with moderate or severe asthma used the portal more frequently (75% were frequent users vs 47% were parents whose child had mild persistent asthma).
Fiks, 2016 [32]	United States	MyAsthma	Descriptive, mixed methods	237 families	Users were more likely to have children aged 6-9 years (*P*=.009), be White (*P*<.001), be privately insured (*P*<.001), have mild persistent or moderate or severe persistent asthma (*P*=.002), be on an asthma controller medication (*P*<.001), and be receiving a greater number of asthma medications at baseline on average than nonusers (*P*<.001). Those with persistent asthma were twice as likely to use the portal versus those with intermittent asthma (2.37% vs 1.25% at hospital practices where these data were available; *P*<.001). Sustained portal users were more likely than one-time users to be Hispanic (*P*=.02), have private insurance (*P*=.02), and be from the Northeast (*P*=.001). Sustained use parents had higher educational levels (*P*=.002). Positively associated with portal adoption in multivariable logistic regression: receipt of a controller medication at baseline (OR 2.0, 95% CI 1.5-2.7), private insurance (OR 2.0, 95% CI 1.3-3.1), lower child age (OR 1.4, 95% CI 1.1-1.9), and greater asthma severity (OR 1.9, 95% CI 1.2-3.0 for mild and OR 1.9, 95% CI 1.0-3.5 for moderate or severe persistent vs intermittent).
Foster, 2019 [43]	United States	Epic MyChart	Observational, hypothesis testing	208,635 tests	Females (5546/13,149, 42.18%) were significantly more likely to activate the portal than males (3897/12,212, 31.91%; *P*<.001). Activation rates were highest for Asian (262/451, 58.1%) and White individuals (8155/20,637, 39.52%) and lower for African American/Black (491/2254, 21.78%; *P*<.001 compared with White), Hispanic/Latino (333/1257, 26.49%; *P*<.001 compared with White), and other individuals (241/762, 31.6%; *P*<.001 compared with White). The activation rate for patients aged 18-70 years was 41.61% (7593/18,246). The overall pattern of radiologic image viewing with respect to age and gender showed similar trends to those described for laboratory testing.
Gordon, 2016 [20]	United States	Kaiser Permanente Northern California patient portal	Observational, hypothesis testing/descriptive, quantitative	231,082 patients/4980 patients	Older seniors (aged 70-74 and 75-79 years) were significantly less likely than those aged 65-69 years to have registered, and to have used the patient portal to send a secure message, view laboratory test results online, or order prescription refills at least once by the end of the year. Slightly over 70% had been diagnosed with a chronic cardiovascular condition, and 90% reported taking at least one prescription medication for a chronic condition.
Gossec, 2017 [59]	France	Sanoia	RCT	320 patients	In multivariate analyses, the only factor related to connecting more than twice to the platform was being a member of a patient association (OR 1.44, 95% CI 1.17-1.77; *P*<.001). In the groups with high and low numbers of connections, the percentages of patient association members were 24.7% and 6.5%, respectively.
Griffin, 2016 [44]	United States	My UNC Chart	Observational, hypothesis testing	2975 patients	Active users had a higher proportion of Caucasian patients, higher Charlson Comorbidity scores, and a higher proportion of patients admitted to an academic medical center than light users.
Huang, 2019 [45]	United States	myPennMedicine (branded version of Epic MyChart)	Observational, hypothesis testing	10,000 patients	Users were more likely to be younger (63.46 years [users] vs 66.08 years [nonusers]; *P*<.001) and have higher income (US $74,172 [users] vs US $62,940 [nonusers]; *P*<.001) than nonusers. The percentage of White race was substantially higher among users (72.77% [4317/5932] [users] vs 52.58% [2139/4068] [nonusers]; *P*<.001). For users, the percentage of payments by commercial insurance was higher (60.99% [3618/5932] [users] vs 40.12% [1632/4068] [nonusers]; *P*<.001) and the percentage of payments by Medicare or Medicaid was lower (Medicare: 34.91% [2071/5932] [users] vs 48.72% [1982/4068] [nonusers]; *P*<.001; and Medicaid: 3.49% [207/5932] [users] vs 10.08% [410/4068] [nonusers]; *P*<.001). The difference in sex between users and nonusers was not statistically significant. No significant difference was found in any provider-level characteristic between the two groups.
Jhamb, 2015 [46]	United States	Free patient portal tethered to an ambulatory EHR^c^	Observational, hypothesis testing	1098 patients	Users were younger and more likely to be non-Black, be married, have private insurance, and have higher neighborhood median household income. Users were less likely to have diabetes, coronary artery disease, or congestive heart failure, but were more likely to have had a kidney transplant. Older age, Black race, unmarried status, Medicaid or Medicare insurance (vs private), and lower neighborhood median household income were associated with not using the portal.
Kipping, 2016 [35]	Canada	Ontario Shores HealthCheck Patient Portal	Observational, hypothesis testing	91 patients	A similar proportion of patients (1756/3158, 55.6%) and portal users (266/432, 61.6%) were female. Age distribution was relatively similar. The majority of users were between 25 and 34 years.
Krist, 2014 [33]	United States	AllscriptsTouchworks EHR	Descriptive, mixed methods	112,893 patients	Older patients were more likely to create a PHR^d^ account as they had chronic conditions.
Laranjo, 2017 [22]	Australia	Portuguese National patient portal	Descriptive, quantitative	109,619 participants	Geographic analysis revealed higher proportions of PHR adoption in urban centers when compared with rural noncoastal districts.
Lau, 2014 [36]	Canada	BCDiabetes.ca	Observational, hypothesis testing	1957 patients	Users tended to be younger (mean difference of 4.28 years; *P*=.06), have lower baseline HbA_1c_ (mean difference of 0.89%; *P*<.01), and have higher baseline weight (mean difference of 7.53 kg; *P*=.06) than nonusers. There was no difference in gender or total follow-up time. Follow-up HbA_1c_ levels tended to be lower in users than nonusers (mean difference of 0.75%; *P*<.01), and users were significantly more likely to have HbA_1c_ of 7% at their last follow-up visit (*P*=.01). No significant differences in LDL^e^ and SBP^f^ were observed between users and nonusers at initial visits and follow-up visits.
Manard, 2016 [37]	United States	Online patient portal	Observational, hypothesis testing	1571 patients	Users were significantly younger (*P*<.001), more often White (*P*<.001), and more often married (*P*<.001) than nonusers. Users were significantly from upper-middle to the highest socioeconomic status compared with nonusers (*P*<.001). Portal use was more common among FM^g^ patients than GIM^h^ patients (*P*<.001), and users were more often high health care utilizers (*P*<.02). Portal use was less common among current smokers (*P*<.001). Users were more likely to have depression (*P*<.01) and lower comorbidity scores (*P*<.001).
Mishra, 2019 [23]	United States	OpenNotes within the HealtheLife patient portal	Descriptive, quantitative	1487 patients	Overall, 90% (n=784) were above 30 years, with 8% between 18 and 29 years; 40% were above 60 years; and 50% were between 30 and 59 years. One participant stated accessing the portal for a minor and another as a family surrogate. Moreover, 92% (n=797) had a college degree or greater, 24% (n=205) had a graduate degree, and 1% (n=12) had less than 12th grade education. Individuals in the 18-29 and >60 years groups were more likely to find the notes helpful. Greater note comprehension was correlated with greater education. Noncollege participants were more likely to access notes “many times” than college participants (*P*=.02).
North, 2014 [38]	United States	Mayo Clinic Health System	Observational, hypothesis testing	2357 primary care patients	The majority of patients sending messages were female, were White, lived locally, and were employed by the Mayo Clinic.
Plate, 2019 [39]	United States	MyChart; Epic Systems Corporation	Observational, hypothesis testing	6426 patients	Overall, 4623 people registered on MyChart logged into the patient portal at least once within 1 year from surgery, and 1803 (28%) patients were not registered users. Active users were significantly more likely to be young, have a healthy ASA^i^ score (ASA 1 or 2), be Caucasian, be married, be employed, be privately insured, and be discharged to home. Patients not using MyChart had a higher ASA score (ASA 3 or 4) and were more likely to be African American, unmarried, and unemployed. Patients without MyChart were more likely to have Medicare or Medicaid insurance and be discharged to a skilled nursing facility.
Portz, 2019 [52]	United States	My Health Manager (Kaiser Permanente Colorado patient portal)	Qualitative descriptive study	24 patients	The mean age was 78 years. Patients were primarily White (12, 80%) and women (12, 80%). Education: high school graduate, 1 (7%); some college graduate, 7 (47%); and college graduate, 7 (47%). Income: <US $30,000, 2 (13%); US $30,000-49,999, 7 (47%); US $50,000-74,999, 2 (13%); >US $75,000, 2 (13%); chose not to answer, 2 (13%).
Powell, 2018 [40]	United States	FollowMyHealth portal	Observational, hypothesis testing	500 patients	No significant relationship between the number of logins and any of the demographic variables; however, when those with zero logins were removed from the model, age, distance separating the patient from his or her provider, and having a diagnosis of heart failure were all significant predictors of portal use (*P*<.05).
Price-Haywood, 2017 [25]	United States	MyOchsner patient portals (Epic System), wearable technology, smartphone mobile apps	Descriptive, quantitative	247 patients	Portal users had higher levels of education, lower rates of inadequate health literacy, and higher rates of using the internet and having an interest in websites or smartphone apps for tracking health. The odds of portal use increased with total eHEALS scores (health literacy scale) and decreased among Black patients.
Riippa, 2014 [56]	Finland	No specific portal	Interventional, other than RCT	876 patients	Patients with a severe diagnosis during the intervention showed the greatest positive change in patient activation (mean change 5.4, SD 8.4). Patients diagnosed 1-2 years ago (mean change 2.3, SD 15.7) and patients with no severe diagnoses (mean change 1.6, SD 13.1) showed a positive change in patient activation.
Ronda, 2014 [26]	Netherlands	Digitaal Logboek	Descriptive, quantitative	1390 patients	Multivariable analysis showed that increasing age and smoking were associated with not using the portal. A higher educational level, treatment by an internist, using insulin, polypharmacy, better diabetes knowledge, and more hyperglycemic episodes were less likely to be associated with not using the portal.
Smith, 2015 [27]	United States	EpicCare	Descriptive, quantitative	534 patients	Significant predictors of registering were as follows: gender (male 65.3% vs female 55.1%), race (White 71.7% vs African American 27.7% vs “other” races 41.7%), education (more educated people were more likely to register), number of chronic conditions (70.9% with zero conditions, 63.2% with one condition, and 50.0% with two or more conditions), health literacy (adequate 72.7% vs marginal 46.4% vs limited health literacy 21.7%).
Sun, 2019 [41]	United States	Epic’s personal health record system	Observational, hypothesis testing	38,399 patients	Almost one-third of patients (n=12,615; 32.9%, 95% CI 32.38%-33.32%) had used the portal for a mean of 2.5 (SD 1.9) years prior to the study period. Portal use was higher on weekdays (*P*<.001). An increase in portal use was observed in response to email reminders. A nonlinear relationship between age and portal use was observed and depended on several other predictors (*P*<.05). Patients living in more rural areas with low income were at lower odds to use the portal (*P*=.02), and this finding also applied to non‐Whites with low income (*P*<.001). More chronic conditions and a higher initial HbA_1c_ value were associated with portal use (*P*=.01).
Tsai, 2019 [28]	United States	Epic’s personal health record system	Descriptive, quantitative	109,200 patients	Active portal users were on average older (49.45 vs 46.22 years) and frequently female (62.59% vs 54.91%). Both the differences in mean age (*P*=.008) and gender (*P*=.04) were significant. There was a bimodal peak in terms of active users, with active users more likely to be in their 30s and 60s. The difference among age groups was significant (*P*<.001). Differences in racial composition, insurance, and language were not significant.
Van der Vaart, 2014 [57]	Netherlands	Medisch Spectrum Twente	Interventional, other than RCT	360 patients	Univariate analyses showed that age, marital status, education level, employment, health literacy, and internet-related characteristics were significantly related to portal use. Nonusers were more often older, single, lower educated, and unemployed. Respondents with higher health literacy were more inclined to login on the portal, and respondents who used the internet more often had more years of experience and perceived their own skills as better.
Wade-Vuturo, 2013 [8]	United States	MyHealthAtVanderbilt patient portal	Descriptive, quantitative	54 patients	Participant age, gender, race, income, and education level were not associated with using SM^j^ to send a message to a provider for any reason or using SM to schedule an appointment.
Wallace, 2016 [58]	United States	MyChart by Epic health record system	Interventional, other than RCT	36,549 patients	Men, non-White patients, and Hispanic patients were significantly less likely to login once, 2 to 23 times, or 24 times than women, White patients, or non-Hispanic patients. Patients with public insurance were less likely to login than those with private insurance across all MyChart usage categories. Patients with income levels 100% of the FPL^k^ were more likely to login one time than those below the FPL level.
Wedd, 2019 [30]	United States	Unspecified patient portal	Descriptive, quantitative	710 patients	Black patients were less likely to use the portal vs White patients among both kidney (Black 57% vs White 74%) and liver (Black 28% vs White 55%) transplant recipients. In adjusted multivariable analyses, kidney transplant recipients were more likely to use the portal if they had higher education. Among liver recipients, patients who were White and had higher education were more likely to use the portal.
Zhong, 2018 [42]	United States	MyUFHealth (also known as MyChart by Epic)	Observational, hypothesis testing	15,659 nonusers and 5494 users	The user group comprised 53.1% patients with more than four chronic problems (vs 40.2% of the matched nonuser group), and had more patients bearing 10 or more chronic problems (18.2% vs 12.2%). Individuals enrolled in the patient portal were mostly middle aged (31-64 years) and female. Married patients were more likely to adopt the portal. Medicare and Medicaid patients, and Black or African American patients were less likely to be adopters. Portal adoption was also associated with the baseline number of active medical problems (*P*<.05).

^a^OR: odds ratio.

^b^RCT: randomized controlled trial.

^c^EHR: electronic health record.

^d^PHR: personal health record.

^e^LDL: low-density lipoprotein.

^f^SBP: systolic blood pressure.

^g^FM: family medicine.

^h^GIM: general internal medicine.

^i^ASA: American Society of Anesthesiology.

^j^SM: secure messaging.

^k^FPL: federal poverty level.

In terms of age, results were not homogeneous. Higher use in older patients (aged >50 years) was described in four studies [18,20,21,33]. In a middle age and elderly cohort, the mean age of users was significantly lower [45]. On the other hand, younger patients were also the major users in disease/specialty-specific cohorts [32,35,46].

A total of 19 studies mentioned the origin and ethnicity of users [8,18,25,27,30-32,34,37-39,42-46,52,58]. White patients were usually the most likely to use the portals described in the different studies [18,27,30-32,37,38,43,45,52]. Two studies found no significant association between ethnicity and portal use [8,28].

Most of the articles found a positive association between female gender and portal use [28,35,38,42,43,58]. Only one study, conversely, found male gender to be a predictor of registering [27]. No statistically significant association between sex and user/nonuser status was found in two articles [36,45].

In a cohort of patients with mental disorders, having schizophrenia or schizoaffective disorders was negatively associated with portal use [31]. Instead, depression was positively associated [37]. Moderate or severe asthma was more linked to portal use [32,60]. Moreover, having a diagnosis of diabetes, hypertension, heart failure, or cardiovascular disease was a significant predictor of portal use [20,40], with one exception [46]. On the other hand, there were contrasting results concerning the association between the number of comorbidities and portal use [27,37,41,42,44].

People living in rural areas were less likely to use patient portals than urban citizens in three studies [22,31,41], while higher education levels were often related to broader use of portals [23,25-27,30,32,52,57]. Only one study showed no significant association [8]. Higher income was also generally associated with portal use [31,37,45,46,58]. Only one study found no association between income and the use of secure messages [8].

Studies conducted in the United States showed that having a private insurance was positively associated with portal use [18,32,39,42,45,46,58], with only one study reporting the absence of this association [28].

Other patient characteristics positively associated with portal use were being a member of a patient association [59] and being admitted to an academic medical center [44].

### Attitudes and Satisfaction

Patient attitudes were evaluated in terms of perceived barriers and facilitators toward portal use. The overall satisfaction was also assessed, and it refers to the extent to which the patient is content about health care. Thirty articles addressed these topics (Multimedia Appendix 4).

Nine articles clearly addressed the barriers to portal use. Some of the main issues were related to perceived or preconceived security concerns [8,48], limited knowledge [26,51], satisfaction with current care [51], paying for the service [54], disinterest in managing one’s own disease [26,57], personal/time constraints and not thinking about accessing the portal [57,61], doubts about the reliability of the patient portal to facilitate a timely and productive message exchange with providers [8], and prior negative experiences with secure messaging [8]. Other barriers were related to population characteristics, such as being a clinician older than 55 years or younger than 35 years and being male [33], and variation in provider availability for online appointment scheduling and response times to medical messages [25]. Limited computer and internet access [20,25,26,51,57], knowledge of technology [25,26,57], security concerns [25,48], and data integration [48] were also negative predictors of portal use.

On the other hand, nine articles specified the elements that facilitated portal use, including improved communication with specialists [22,48,60]; availability of information that led to an increased awareness of the health status [19,22,32,51,60] and tracking of disease control [32]; time-saving, convenient, and easy to use elements [19]; accuracy, timeliness, usefulness, and convenience of the functionalities included in the portal [22,50]; availability of surrogates (ie, daughter or family member) to act as intermediaries [51]; active involvement of the practice/staff in the promotion of the portal (ie, team approach strategy to engage staff in notifying patients) [33]; and active training of the patient for portal use [61]. Concerning satisfaction, patients were generally satisfied with the portals [8,17,21,29,49,50,59-61].

## Discussion

Due to the considerable amount of literature published on the topic of patient portals, the aim of this review was to provide evidence and to gather information systematically. Similar to the review published in 2013 by Goldzweig et al [4], the outcomes were grouped into four aspects, namely, health outcomes and adherence, health care efficiency, patient characteristics, and attitudes and satisfaction, and showed nonunique results in terms of benefits brought by patient portals concerning patient experience and health. The functionalities available in the portals described by Goldzweig et al and our review were likewise the same.

Positive results were described relating to the enhancement of preventive behaviors [45], changes in chronic condition control with higher control of diabetes parameters [34,36], and asthma flares [60]. However, conflicting results were described concerning blood pressure control [34,37], mental health conditions [35,54,55], and medication adherence [32,57]. A possible explanation of these results is that these particular studies involved patients (often with a low sample size) who utilized portals for a short period of time, preventing them from having a possible consistent outcome. Similarly, concerning health outcomes, Goldzweig et al found generally positive results, which documented improvement in patients’ disease control and maintenance [4], even though many of the included studies in our review did not find a significant difference between portal users and nonusers. Adherence to therapy was always improved [4] similar to that in this review.

The number of clinical and ED visits [28,32,39,54,60] and hospital readmissions [39,44,54,60] did not always decline in patients using portals. The number of missed appointments decreased [35,42], but this finding was not always significant [28,42]. The nondeclining trends of hospitalizations and outpatient visits as a consequence of portal use are concordant in the two studies, although different findings were described. Indeed, different from our review, in which we found more often a decrease in the utilization of in-person services, the review by Goldzweig et al reported that most of the studies found an increased number of outpatient visits and hospitalizations. As patient portals are normally designed to reduce inappropriate health care utilization, this might be partially explained by the fact that provider and patient adaptations to the patient portals have evolved over time.

The studies differed also in terms of the population included, with conflicting results in portal adoption and age, gender, ethnicity, kind of disease, and number of comorbidities. Generally, having a higher income [31,37,45,46,58] and having a private insurance were associated with increased portal use [18,32,39,42,45,46,58].

The main barriers faced in using the portals were (1) user-related issues, such as time constraints [57,61], disinterest in managing the disease [26,57], and limited digital knowledge [26,51]; (2) clinician-related issues, such as age [33] and attitude toward the portal [33]; and (3) technology-related issues, such as limited internet access [25,26,51,57] or ability to use technology [25,26,57] and security concerns [25,48]. Privacy and security concerns, and ability to use technological appliances and systems were the most important barriers to utilization described in the studies retrieved by Goldzweig et al [4]. Indeed, other recent reviews on patient attitudes highlighted that privacy and security problems are the main barriers to the use of patient portals. These barriers are as evident in elderly patients (in whom there is also a limitation of portal use related to age) as in younger patients [62,63]. Moreover, technical problems due to patient capacity and difficulties in using the portal also represent important barriers. Thus, correct and adequate information on safety issues and education on the technical use of the portal represent the best facilitators. Furthermore, engaging patients and making them realize that the portal represents a useful tool to support the management of their pathology (especially for chronic diseases) without replacing the doctor-patient relationship are important to encourage the use of digital portals [13,62,64]. In addition, technical improvements in the usability of portals could increase patient enrollment.

Security problems are complex issues that must be considered in any part of medical care. The use of information technologies in health care that can be accessed by multiple types of users (physician, patient, caregiver, and hospital administrative staff) represents the basis of the discussion about computer security [65]. Indeed, uncertainties about security of clinical data might hinder adoption of systems by both hospitals and patients [48]. Patients expressed concern about their privacy and the privacy of their family members, and asked for further information about confidentiality, as vulnerable data might be accessed by external providers, such as insurance providers, who are the main actors of health care access in many countries [47].

The facilitators retrieved were the prompt availability of health information that caused an increase in the awareness of the health status [19,22,32,51,60], improved communication with health care professionals [22,48,60], and the accuracy, timeliness, usefulness, and convenience of the functionalities included in the portal [22,50]. In this latter argument, some functionalities of the portal were found to be more useful than others, including laboratory tests and imaging [23,24,29,43,52], medical notes [23,26], messaging with providers [52], medication refill [52], and current medication list [29]. Generally and as observed by Goldzweig et al [4], patients declared being satisfied with the use of digital tools [8,17,21,29,49,50,59-61].

Despite the considerable number of studies included, the high heterogeneity in terms of outcomes and described portal functionalities did not allow us to perform meta-analyses and to draw generalizable and strong conclusions concerning the utility of the unique features of the portals.

Technological and digital innovations in health care could contribute to achieving the health system goals of equity, efficiency, accessibility, quality, and sustainability, if they are purposefully designed and cost-effectively implemented. When designing a new patient portal or a new functionality, developers and providers should always consider to which health care need they are trying to respond and if other nondigital interventions may be more effective or as effective at a lower cost.

Moreover, the adoption of a new technology is a complex process, depending on the content and the context in which it is introduced. As an example, our review demonstrated that it is feasible to achieve better medication adherence in chronic disease patients through portal use, and highlighted the main facilitators (eg, prompt availability of reliable information and accessibility of communication with disease specialists) and barriers (eg, security and usability concerns, and limited digital knowledge) to portal use. Keeping in mind these contextual factors could ease the difficult task of identifying the best digital tool for a specific population.

Before designing or implementing a new tool, it can be useful to analyze the ideal conditions needed for the adaptation, transfer, absorption, up-scaling, and enhancement of digital technologies. By ideal conditions, we basically mean a situation where the new technology has demonstrated effectiveness in trials or pilots, the provider is committed to guarantee continuous improvement in user accessibility and usability, and the main barriers in the target population are given due consideration. In the absence of these conditions, satisfactory results may be difficult to reach or may take many years to be observed.

The benefits of digitalization cannot be taken for granted and the use of technology does not always lead to an improvement in patient care and health system performance; thus, there is a need for evidence, which is, to date, scarce. The identification of a set of main features with proven efficacy for a patient portal is a useful starting point for the development and implementation of patient-oriented portals. Further studies should be conducted in different aspects of digitalization in health care. None of the studies retrieved analyzed the cost-effectiveness of portal use. Similarly, none of the studies compared the portals to each other, which could be interesting to point out the best practices and features.

Even though a patient portal is not a new concept, its real utilization and implementation are still far from optimal, and it seem to be still considered a “future technology.” It is important to adapt the portal functions to the needs and capacities of patients, in order to facilitate the use of this technology and improve its dissemination. In particular, overcoming ethnic and literacy barriers to portal use represents a fundamental goal to create more equitable, effective, and safe health care systems.

## Appendix

Multimedia Appendix 1 Search string.

Multimedia Appendix 2 Functions and details of patient portals.

Multimedia Appendix 3 Qualitative descriptions of the portals and/or features studied in the included articles.

Multimedia Appendix 4 Summary of the findings on patient attitudes and satisfaction.

## Abbreviations

ED: emergency department
EMR: electronic medical record
ER: emergency room
RCT: randomized controlled trial
