# Supplementary material for: The Impact of Digital Patient Portals on Health Outcomes, System Efficiency, and Patient Attitudes: Updated Systematic Literature Review
Source: J Med Internet Res. 2021 Sep 8;23(9):e26189. doi: 10.2196/26189 (PMC8459217; doi:10.2196/26189)
Supplement: Multimedia Appendix 1 [file jmir_v23i9e26189_app1.docx]

**Multimedia Appendix 1.** Search string.

(Electronic Health Records [MeSH Terms] OR electronic health record OR electronic health records OR electronic medical record OR electronic medical records OR digital health record OR digital health records OR digital medical record OR Digital Medical Records OR computerized alerts OR computerized reminder OR computerized reminders OR computerized alert OR computerised alerts OR computerised reminder OR computerised reminders OR computerised alert OR (Medical Records Systems, Computerized[MeSH Terms])) AND (patient portal* OR family portal* OR citizen portal* OR consumer portal* OR personalised portal* OR personalized portal* OR caregiver portal* OR app OR apps OR application OR applications OR patient platform* OR family platform* OR citizen platform* OR consumer platform* OR personalised platform* OR personalized platform* OR caregiver platform*) AND (Patient OR Patients OR Family OR Families OR Citizen* OR Consumer* OR personalised OR personalized OR caregiver).
