# Supplementary material for: The Impact of Digital Patient Portals on Health Outcomes, System Efficiency, and Patient Attitudes: Updated Systematic Literature Review
Source: J Med Internet Res. 2021 Sep 8;23(9):e26189. doi: 10.2196/26189 (PMC8459217; doi:10.2196/26189)
Supplement: Multimedia Appendix 2 [file jmir_v23i9e26189_app2.docx]

**Multimedia Appendix 2.** Functions and details of patient portals.

| **Portal** | **Author, year** | **Test results** | **Secure messaging** | **Notes, summaries** | **Educational materials** | **Medication renewal** | **Schedule appointments** | **Patient upload, diary** | **Online reminders** |
| --- | --- | --- | --- | --- | --- | --- | --- | --- | --- |
| Allscripts | Krist, 2014 | x |  | x | x |  |  |  |  |
| BCDiabetes | Lau, 2014 | x | x |  | x |  |  |  |  |
| Cerner web portal | Wedd, 2019 | x | x |  |  | x |  |  |  |
| Connect2care | King, 2017 | x | x | x |  |  | x | x | x |
| Digital Logboek | Ronda, 2014 | x | x | x |  |  |  | x | x |
| eCare | Stein, 2018 | x |  | x |  |  |  |  |  |
| Epic | Jhamb, 2015  Sun, 2019 | x | x | x | x | x | x | x | x |
| EpicCare | Chan, 2018  Smith, 2015  Ancker, 2019 | x | x | x |  | x | x | x |  |
| EpicCare MyAsthma | Fiks, 2015  Fiks, 2016 | x |  | x | x | x | x | x | x |
| Epic MyChart | Foster, 2019  Plate, 2019  Schultz, 2018  Wallace, 2016  Zhong, 2018  Tsai, 2019 | x | x | x | x | x | x | x | x |
| Epic MyChart (Variant of) | De Jong, 2018  Huang, 2019  Wallace, 2016 | x | x |  |  |  |  | x |  |
| Epic MyOchsner | Price-Haywood, 2017 | x |  | x |  |  | x |  | x |
| Epic My UNC Chart | Griffin, 2016 | x | x | x |  |  |  | x |  |
| FollowMyHealth | Powell, 2018 | x | x | x | x |  |  | x |  |
| HealtheLife patient portal | Mishra, 2019 | x | x |  | x | x |  | x |  |
| In-house portal | Byczkowski, 2014 | x | x | x |  |  |  | x | x |
| Journalen | Moll, 2018 | x |  | x |  |  |  | x | x |
| Kaiser Permanente Northern California | Gordon, 2016 | x | x |  | x |  |  |  |  |
| Mayo Clinic | North, 2014 | x | x | x | x |  | x | x |  |
| Medisch Spectrum Twente Patient Portal | Van der Vaart, 2014 | x |  | x | x |  |  | x |  |
| MyHealthAtVanderbilt | Wade-Vuturo, 2012 | x | x |  | x | x | x | x |  |
| My HealtheVet | Abel, 2018  Jahn, 2018  Mishuris, 2014  Turvey, 2014 | x | x | x | x | x | x | x | x |
| My Health Manager (Kaiser Permanente Colorado) | Portz, 2019 | x | x |  | x | x | x |  |  |
| Ontario Shores HealthCheck Patient Portal | Kipping, 2016 | x |  | x | x | x | x | x |  |
| Patients Know Best | Bidmead, 2016 | x |  | x |  |  |  | x |  |
| Patient Online | Abd-Alrazaq, 2019 | x | x | x |  | x | x |  |  |
| PatientSite | Bajracharya, 2019 | x | x |  | x | x |  |  |  |
| Portuguese Personal Health Record | Laranjo, 2017 | x |  | x |  |  | x |  |  |
| Sanoia | Gossec, 2017 | x | x | x |  |  |  | x |  |
| Seva | Quanbeck, 2018 |  |  | x | x |  |  | x |  |
|  | Jackson, 2017 |  |  |  | x |  |  | x |  |
|  | Manard, 2016 |  | x |  |  |  | x | x |  |
|  | Riippa, 2014  Riippa, 2015 | x | x | x | x |  |  |  | x |
