# Supplementary material for: The Impact of Digital Patient Portals on Health Outcomes, System Efficiency, and Patient Attitudes: Updated Systematic Literature Review
Source: J Med Internet Res. 2021 Sep 8;23(9):e26189. doi: 10.2196/26189 (PMC8459217; doi:10.2196/26189)
Supplement: Multimedia Appendix 3 [file jmir_v23i9e26189_app3.docx]

**Multimedia Appendix 3.** Qualitative descriptions of the portals and/or features studied in the included articles.

| **1st Author, Year Country** | **Name of the portal** | **Description of the portal** |
| --- | --- | --- |
| **Abd-Alrazaq, 2019 England** | Patient Online. | Program launched online by the National Health Service in England launched. It requires general practices (GPs) to provide patients with Web-based services, such as booking appointments, requesting prescription refills, and viewing summary information from GP records. GPs use one of the following systems to provide their patients with the abovementioned services: SystemOnline, Patient Access, Patient Services, The Waiting Room, Engage Consult, and Evergreen Life or i-Patient. |
| **Abel, 2018 USA** | My HealtheVet (MHV) and Clinical Video Telehealth (CVT). | MHV portal allows users to create and maintain a personal health record by using secure messaging, web-based prescription refills, access to information in VHA health record (i.e. laboratory results, clinical progress notes, discharge summaries, and medication lists), and tracking of personal and self-reported health information using a variety of tools (i.e. food, activity and allergy journals, family health history). CVT is a two-way interactive and synchronous videoconferencing. |
| **Ancker, 2019 USA** | EpicCare electronic medical record and its integrated portal, the Weill Cornell Connect. | The provider selects patients for whom flowsheet is appropriate; patients cannot initiate the use of flowsheets. After a physician enables the flowsheet, the patient can use it to upload data securely. The portal is accessible either through a web browser or a smartphone app. Each blood glucose value is labeled with the time, and the patient can also enter insulin dose and time of administration, and free text notes. Patients may upload several values per day. The physician can view the data. |
| **Bajracharya, 2019 Israel** | PatientSite. | The computer-based family history module, available to all Beth Israel Deaconess Medical Center patients with access to the portal PatientSite, consists of 39 primary questions about the incidence of familial diseases and questions that enable patients to specify relationships to family members with histories of familial diseases. |
| **Bidmead, 2016 England (UK)** | Patients Know Best (PKB). | Patient portal with integrated, patient-controlled digital care records. PKB is an internet based, provider-tethered information exchange system. It allows communication via video-conferencing and through secure messaging. Patients can upload information and access lab results and letters from providers. Patients can permit access to others. PKB combines portal access with an electronic, integrated, PHR. |
| **Byczkowski, 2014 USA** | - | In-house web-based patient portal |
| **Chan, 2018 USA** | MyChart (EpicCare, Verona, WI). | Patient portal that allow secure messaging with practice providers, to request medication refills and to view appointments, test results, after-visit summaries, medical problem lists, allergies, and immunizations. |
| **De Jong, 2018 Netherlands** | Virtual Outpatient Clinic (VOC). | Multicomponent digital health cloud service that integrates data sharing, collection, and communication to facilitate patient-centered care in combination with a hospital patient portal and care professionals. The VOC consists of 6 digital tools that facilitate self-monitoring (blood pressure, weight, and pain) and communication with peers and providers (chat and videoconferencing) connected to a cloud-based platform and the hospital patient portal to facilitate access to (self-collected) medical data. The VOC consists of 2 measuring devices and 4 smartphone apps, of which 2 collect health data and 2 facilitate communication. The VOC is a combination of hospital’s patient portal system (PPS) and personal health record (PHR) and consists of multiple health-monitoring tools, in which data storage and presentation are integrated and can be accessed by both patients and health care professionals. |
| **Fiks, 2015 USA** | MyAsthma. | Clinical interface within MyChart. Features: identification of parents’ concerns and goals for asthma treatment; monthly tracking of symptoms, medication side effects, and progress toward goals; asthma educational content including videos; and access to the child’s asthma care plan. |
| **Fiks, 2016 USA** | MyAsthma. | It provides educational material; it enables sharing of families’ treatment concerns, goals, asthma symptoms, medication adherence, and side effects with the primary care clinical team; it tracks asthma control over time; and it provides decision support to both families and clinicians regarding asthma control and side effects. |
| **Foster, 2019 USA** | MyChart | Epic's personal health record system. |
| **Gordon, 2016 USA** | Kaiser Permanente Northern California (KPNC) patient portal. | It allows to communicate with health care providers and specialists through secure messaging, to view laboratory test results, to order and pay for prescription refills, to view and schedule appointments for primary care and vision care, to check preventive care status and prescribed medication list, to complete online health questionnaires, to use patient/health education programs not available to the public, and to download variety of forms for use within Kaiser Permanente. |
| **Gossec, 2017 France** | Sanoia. | Online interactive electronic e-health platform developed to allow patient self-assessment and self-monitoring. |
| **Griffin, 2016 USA** | My UNC Chart. | The patient portal gives patients controlled access to medical records through a web browser or mobile application |
| **Huang, 2019 USA** | MyPennMedicine | A branded version of Epic MyChart. It provides users with real-time information about medical records and test results, prescriptions, and appointments and other important health information |
| **Jackson, 2018 USA** | - | - |
| **Jahn, 2018 USA** | My HealtheVet. | The secure messaging system connects providers interacting with the VA's EHR system to patients interacting with their PHR. |
| **Jhamb, 2015 USA** | - | Free patient portal tethered to an existing ambulatory EHR |
| **King, 2017 Canada** | Connect2care. | It gives electronic access to medical records, online appointment cancelling and booking features, transparent and timely access to clinical documentation, and e-messaging to connect with care providers. |
| **Kipping, 2016 Canada** | Ontario Shores HealthCheck Patient Portal. | It allows patients to view EMR information; to request medication renewals; to view outpatient appointments generated through Meditech’s “Community Wide Scheduling” module; to update demographic or contact information; to access educational materials; communication between patients and physicians and/or interprofessional outpatient clinician team members; and flexibility for future development and iterations to meet evolving needs of patients and clinicians. |
| **Krist, 2014 USA** | AllscriptsTouchworks EHR and commercial portal that only provides secure patient messaging. | The two tools are not integrated. Allscript is an Interactive Preventive Health Record, IPHR. Patients create an account that links the system to their clinician’s EHR and complete a health risk assessment of patient-reported information (i.e. health behaviours) and information poorly recorded in EHRs (i.e. family history, surgical history). IPHR generates a personally tailored list of preventive and chronic care recommendations and educational material. Patients can view all laboratory results with explanatory messages from their clinicians. |
| **Laranjo, 2017 Australia** | Portuguese National patient portal. | PHR is connected to a national shared record integrating EHR data from multiple NHS providers. The PHRs were implemented in an opt-in model (i.e. people had to actively sign up if they wanted to have an account), and the national shared records were created in an opt-out model, which means that there was implied consent for the creation of a record for each person. PHR allowed patients to input health information (i.e. health problems, chronic medication, and biometric measurements) and book primary care consultations. |
| **Lau, 2014 Canada** | BCDiabetes.ca. | It provides a library of medical education documents, a journal entry application, access to up-to-date personal laboratory values, and a secure email/messaging system. Only the patients can use and view their own journal entries. Laboratory values are provided in a chronologic matrix form and also in a detailed form; mouse-over simple explanations for common laboratory parameters are provided. |
| **Manard, 2016 USA** | - | Online patient portal that allows patient to access laboratory results, vital signs, diagnosis, medication fill requests, and a message interface to communicate with providers via the Internet. |
| **Mishra, 2019 USA** | OpenNotes whithin HealtheLife patient portal. | HealtheLife web-based tool includes patient education, secure messaging, medication refills, laboratory results, pethology results, radiology results, and provider notes. |
| **Mishuris, 2015 USA** | My HealtheVet (MHV). | It allows patients to access health information and communicate with the healthcare team. |
| **Moll, 2018 Sweden** | Journalen for patient-accessible EHR (PAEHR). | Accessible through Web via the national patient portal. The PAEHR service accesses the EHR information through a national health information exchange platform. Hence, patients have one access point to all their health record information regardless of how many health care providers they have visited, and which EHR system their health care providers use. |
| **North, 2014 USA** | Mayo Clinic Health Sistem. | Portal services allowed patients to send messages to the healthcare team as secure message or electronic visit (“e-visit”). A secure message was an unstructured message with free-text subject and body fields like an e-mail, addressing ongoing care issues. The e-visits were used for new symptoms. The e-visit led patients through a computer-directed interview, resulting in a structured message with information pertinent to the new symptom. Providers had the option of responding to e-visits asynchronously by text (secure message) or telephone. |
| **Plate, 2019 USA** | MyChart; Epic Systems Corporation. | The MyChart portal allows patients to view clinic visit notes, laboratory results, and imaging results, and to send secure messages to the primary providers. These messages are received in the providers inbox in the EMR system. Patient access to MyChart is gained through a website or smart phone application. |
| **Portz, 2019 USA** | My Health Manager. Kaiser Permanente Colorado’s patient portal | Features: Appointment Center (schedule or cancel appointments); My Medical Record (view test results, immunization records, medical problem list, and care plans); Pharmacy Center (manage prescriptions and order medications); Health Guides and Health Management Tools (Access to health resources and self-management tools for diet, exercise, smoking cessation, and disease specific care, also personalized assessments and health self-management tools); Message Center (email with their provider); Recently added features (e-visit and provider chat functions for non-emergent questions and visits) |
| **Powell, 2018 USA** | FollowMyHealth portal | Direct access to portions of the electronic health record (EHR) including medications, allergies, problem lists, and visit summaries, via the web or mobile device. It also allows patients to enter data (i.e. weight, blood glucose readings) directly into the EHR and interact with a provider via secure messaging. |
| **Price-Haywood, 2017 USA** | MyOchsner | Patient portals (Epic System), wearable technology, and smartphone mobile applications |
| **Quanbeck, 2018 USA** | Seva. | It provides patients a discussion board; interactive modules to teach problem-solving, self-regulation, and other skills; tools for coping with cravings and high-risk situations; and health tracking. For clinicians, Seva provides a Web portal with a Clinician Report containing longitudinal information generated by patients’ self-reported data about their substance use and well-being. |
| **Riippa, 2014 Finland** | - | - |
| **Riippa, 2015 Finland** | - | The patient portal contains the electronic care plan, and access to patient records (provided and maintained by the healthcare provider), laboratory results with statements from a healthcare professional, vaccination history, and electronic messaging. Diagnoses, medicines, and laboratory results are linked to relevant additional information in the online medical information service, Health Library, administered by The Finnish Medical Society, Duodecim. |
| **Ronda, 2014 Netherland** | Digitaal Logboek. | Access to diabetes-specific medical records, including information provided by their physician during medical consultation (i.e. physical examination, laboratory results, problem lists, and treatment goals). Laboratory results are accessible as soon as the laboratories report them in advance of a medical consultation. The portal also provides access to general diabetes information and to an overview of all personal diabetes-related examinations and consultations needed and/or scheduled. Patients can import and upload the glucose levels measured at home and contact their physician or diabetes nurse through secured electronic messaging. |
| **Schultz, 2018 USA** | MyNemours. | The portal is based on EPIC's MyChart and available via internet or phone application. It provides access to testing results, problem lists, discharge instructions, letters, medications/allergies, scheduling/appointments, billing, and direct provider messaging. Outpatient results become available four days after finalization (i.e. automatic release) or when reviewed by the ordering physician (i.e. physician release). Inpatient results are automatically available within one day. Certain “sensitive” results (i.e. testing for sexually transmitted diseases) are never released. |
| **Smith, 2015 USA** | EpicCare. | - |
| **Stein, 2018 USA** | eCare. | It allows access to discharge summaries after hospitalization. |
| **Sun, 2019 USA** | Epic's personal health record system. | - |
| **Tsai, 2019 USA** | MyChart | Epic's personal health record system. |
| **Turvey, 2014 USA** | My HealtheVet - Blue Button. | Blue Button is a feature of My HealtheVet patient portal. It allows to access EHR components, such as past and future appointments, problem lists, allergies, medications, laboratory results, procedures, vitals, and immunizations. With Blue Button access, patients can view, download, or print information to share with trusted others. |
| **Van der Vaart, 2014 Netherlands** | Medisch Spectrum Twente. | - |
| **Wade-Vuturo, 2013 USA** | MyHealthAtVanderbilt (MHAV) patient portal. | It allows patients to view EHR data, use Secure Messages to communicate with providers, manage medical appointments and bills, and perform other tasks. All office visits scheduled through the portal are managed via SM, and patients can send a message to their providers for any other purpose. SM is a closed-loop process: patient-initiated messages are dealt with by clinic groups and thus may be answered by a clinic staff member, nurse, or patients’ physician depending on the message content. |
| **Wallace, 2016 USA** | MyChart | Epic's personal health record system. |
| **Wedd, 2019 USA** | - | Institutional tethered online patient portal |
| **Zhong, 2018 USA** | MyUFHealth (also known as MyChart® by Epic®). | Electronic patient portal that provides secure and convenient way to access portions of medical records (i.e. released test results, after visit summary), to communicate with the clinical service providers using secure messaging, to request prescription refills, and to manage outpatient appointments. |
