# Supplementary material for: The Impact of Digital Patient Portals on Health Outcomes, System Efficiency, and Patient Attitudes: Updated Systematic Literature Review
Source: J Med Internet Res. 2021 Sep 8;23(9):e26189. doi: 10.2196/26189 (PMC8459217; doi:10.2196/26189)
Supplement: Multimedia Appendix 4 [file jmir_v23i9e26189_app4.docx]

**Multimedia Appendix 4.** Summary of the findings on patient attitudes and satisfaction.

| 1st Author, Year Country | Health information technology; study design; sample size | Main findings |
| --- | --- | --- |
| Abd-Alrazaq, 2019 England | Patient Online (NHS England); descriptive, quantitative; 624 patients | Adoption rate of Patient Online was 18·9% in April 2017 and 24·4% in April 2018. Behavioural intention (BI) was significantly influenced by performance expectancy (PE; beta = 0·57, P < 0·001), effort expectancy (EE; beta = 0·16, p<0·001), and perceived privacy and security (PPS; beta = 0·24, P < 0·001). Facilitating conditions (FC) and BI significantly influenced use behaviour (UB; beta = 0·25, P < 0·001 and beta = 0·53, P < 0·001, respectively). PE significantly mediated the effect of EE and PPS on BI (beta = 0·19, P < 0·001 and beta = 0·28, P = 0·001, respectively). Age significantly moderated 3 paths: PE→BI, EE→BI, and FC→UB. Sex significantly moderated only the relationship between PE and BI. A total of 2 paths were significantly moderated by education and internet access: EE→BI and FC→UB. Income moderated the relationship between FC and UB. |
| Ancker, 2019 USA | Blood glucose flowsheet (EpicCare and Weill Cornell Connect portal); observational, hypothesis testing; 53 patients | In an academic multispecialty practice with high rates of patient portal use, adoption of a PGHD data upload function has been slow. Over 4 years, 16 providers tried it, and 53 established patients uploaded three or more values. |
| Bajracharya, 2019 Israel | PatientSite (Beth Israel Deaconess Medical Center); descriptive, qualitative; 97,781 patients | 5 main themes: (1) patient empowerment (n = 75); (2) anticipated value for future risk prediction (n = 121); (3) concerns about validity (n = 34); (4) privacy concerns (n = 40); and (5) reflections on patient-computer dialogue (n = 273). Feedback from clinicians has been positive regarding having the data at hand to prompt further discussion and clarification as needed. |
| Bidmead, 2016 England (UK) | Patients Know Best (PKB); descriptive, qualitative; 56 patients | For patients, the system was a source of support and facilitated communication with specialists.  Main barriers to adoption: concerns over security, risk averse attitudes of users (perceptions and pre-conceptions), problems with data integration. |
| Byczkowski, 2014 USA | Unspecified in-house web-based patient portal; descriptive, quantitative; 530 patients | Information was useful, accurate, and timely. The portal was easy to use and easy to learn, more than half of the parents never had difficulties using the portal. Portal rating by parents: excellent (15%), very good (53%), good (24%), or fair or poor (8%). |
| Chan, 2018 USA | MyChart (EpicCare); descriptive, quantitative; 17,699 patients | Patients with resident primary care providers (PCPs) had lower odds of using the portal compared to those with attending PCPs (OR = 0·54, 95% CI 0·50–0·59). |
| De Jong, 2018 Netherlands | Virtual Outpatient Clinic (VOC); descriptive, quantitative; 20 patients | Participants successfully used all options of VOC and were positive about different tools and integral availability of information, reporting an increased awareness of health status.  Facilitators for using the VOC outweighed barriers (30 vs 4, respectively). VOC saved time, was convenient, easy to use. VOC could facilitate exchange of data, monitoring, and virtual consultations, and help in reducing the number of time-consuming hospital visits. |
| Fiks, 2015 USA | MyAsthma (Clinical interface in MyChart); RCT; 60 families of children | A trend toward improved quality of life in terms of daytime symptoms and functional limitations was observed for children receiving the intervention but results were not statistically significant nor were there significant changes in parent activation. For 92% MyAsthma made it easier to care for child’s asthma by facilitating communication, centralizing asthma information, and increasing awareness of the importance of asthma management. No significant differences between study arms in satisfaction with asthma care. In the intervention group, 22 of the 24 parents reported that MyAsthma made it easier to care for their child’s asthma, and that they were satisfied with the portal. |
| Fiks, 2016 USA | MyAsthma; descriptive, mixed-methods; 237 families | Qualitative methods underscored the importance of coordinated practice workflows, including practice responsiveness to portal surveys to implementation success. Parents, especially those with children with uncontrolled asthma, were motivated to continue using the portal because it facilitated a better understanding and tracking of asthma. |
| Foster, 2019 USA | Epic MyChart; observational, hypothesis testing; 208,635 tests | 8·91% of laboratory tests (18573/208635) and 8·97% (2019/22504) of the reports from radiologic imaging studies were viewed. MRI studies were reviewed at higher rates (329/2796, 11·77%) compared with CT scans (859/8957, 9·59%) and x-rays (911/11,750, 7·75%; P < 0·001 for MRI compared with either CT scan or x-ray for all ED patients). MRI studies (brain and spine imaging) and CT scans (abdomen/pelvis, chest, and head/neck) constituted 8 of the 10 tests in the patient portal with the highest frequency of viewing, with some studies viewed by over 30% (eg, MRI cervical spine with contrast: 25/73, 34%).  Wide variability in how fast results were accessed: laboratory results (mean 17·1, SD 42·7 days; median 2·3 days; minimum 0·9 min, maximum 359 days) and radiology reports (mean 24·0, SD 48·3 days; median 3·7 days; minimum 0·9 min, maximum 336 days). Compared with radiology results, laboratory tests tended to be more frequently viewed in less than 1 week (P < 0·001). Nearly half of all results accessed in the patient portal are reviewed within 72 hours of release (laboratory results: 9904/18,573, 53·32%; radiology reports: 971/2019, 48·1%). Laboratory results and radiology reports viewed after 2 weeks constituted 19·93% (3701/18,573) and 971/2019, 48·1%, respectively, of the total views. |
| Gordon, 2016 USA | Kaiser Permanente North California patient portal;  observational, hypothesis testing; 231,082 patients  descriptive, quantitative; 4,980 patients | Barriers: (1) access to digital technology devices declined with increasing age, and across all age groups, black, Latino, and Filipino seniors were significantly less likely than non-Hispanic white seniors to have these digital devices; (2) ability to use the Internet to get health information from websites or to communicate with others significantly differed by race/ethnicity and age; (3) ability to use email. |
| Gossec, 2017 France | Sanoia; RCT; 320 patients | Changes in perceived quality of care were different between the groups: mean (SD) changes from baseline to 12 months were 8·2 (1·7) to 8·3 (1·6) (delta = +0·06 [1·44]) vs. 8·2 (1·6) to 7·8 (1·9) (delta = −0·42 [1·63]) in the Sanoia vs control group (P = 0·02).  RAID (RA Impact of Disease) changes did not differ between groups; both groups saw an improvement in RAID score at 12 months; a further improvement was seen in the coping component of the RAID for the Sanoia group although differences were not statistically significant. Mean (SD) PEPPI (Perceived Efficacy in Patient–Physician Interactions) scores at baseline and 12 months were 38·6 (8·2) and 39·2 (8·0) (delta = +0·60 [5·52]) vs 39·7 (7·3) and 38·8 (8·0) (delta = −0·91 [6·08]) in the Sanoia and control group, respectively (p = 0·01). Total PEPPI-5 scores range 0-50; higher scores represent higher perceived self-efficacy in patient-physician interactions. Satisfaction was high among the 110 patients still followed-up: mean 1·5 (SD 1·5), median 1. Although mean satisfaction with the platform was very high (1·46 [1·52]), 41 patients (25·7%) never accessed Sanoia. |
| Jackson, 2018 USA | Electronic patient information portal; descriptive, quantitative; 21,275 patients | Patients with and also without diabetes were positive about potential benefits of having access to their doctor’s notes with few concerns. Patients with diabetes had consistently higher positive perceptions of OpenNotes than patients without diabetes and also had lower or similar perceptions about the risks. |
| Jahn, 2018 USA | My HealtheVet; descriptive, qualitative; 29 participants | Patients (n = 10) overall satisfaction: 6·0 (IQR: 3·8–6·6). Great variation in satisfaction for new vs experienced patient users of secure messaging (6/7 on Likert scale evaluation) |
| King, 2017 Canada | Connect2care; descriptive, qualitative; 18 parents | Little evidence that the portal led to greater involvement in care process, improved ability to express concerns to providers or enhanced relationships with providers, or reduced number of in-person visits. Facilitators: accuracy and convenience of the client health record, usefulness, timeliness, and adequacy of portal messaging, willingness to invest effort in the intervention process, and convenience in accessing care or services. Moderate degree of perceived usefulness of and satisfaction with the EHR and e-messaging features. |
| Krist, 2014 USA | AllscriptsTouchworks HER; descriptive, mixed-methods; 112,893 patients | Facilitators. Although a range of passive (eg, websites, telephone hold messages, kiosks) and active (eg, distributing cards, brochures) implementation strategies, certain innovations adopted by practices may account for the marked increases. Increased adoption was seen after team approach strategy to engage staff in notifying patients and after availability of laboratory results. Small to medium-sized primary care practices can effectively engage patients to use patient portals by integrating promotion into routine care. A key factor influencing IPHR use was having a comorbid condition.  Barriers. IPHR use was less likely when clinicians were older than 55 years, younger than 35 years, or male. IPHR uptake was lower among smaller practices and practices that relied primarily on clinicians to notify patients about the IPHR, that sent IPHR clinical summaries to the clinician rather than medical records or nursing staff, and that did not use the IPHR to inform patients of laboratory results. |
| Laranjo, 2017 Australia | Portuguese National patient portal (PHR); descriptive, quantitative; 109,619 patients | Facilitators. Given that PHR adoption by patients is influenced by their providers’ endorsement, clinician involvement in the design, development, and implementation of PHRs seems crucial for the success. Continued adoption of PHR will depend on the availability of features that are valuable for patients, such as communication with providers, access to medical records, and administrative functionalities such as prescription refills. |
| Mishra, 2019 USA | OpenNotes whithin HealtheLife patient portal; descriptive, quantitative; 1487 patients | 957/1,122 (85%) individuals reported to have reviewed at least one clinic visit note. Of 896 individuals, 37% (334) looked at their notes once after every visit, 25% (223) looked at notes many times after a visit, and 30% (265) looked at visit notes selectively only if they had questions about the visit. Of the 889 participants, 63% (563) found clinical notes to be always helpful, whereas 35% (309) found clinical notes useful sometimes, and 2% never found them useful. 66% (587) of 886 participants found the clinical plan to be useful, whereas 45% (398) found medications in the note to be helpful. 44% (387) found it useful to remind them when they should come back. 79% (703) found laboratory test results useful. 83% (726) thought OpenNotes helped them take care of themselves better. 94% (820) noted no change or improvement in their worry level.  Out of the 884, 56% reported to understand clinical notes completely, 32% (287) most of it, 92 (10%) “some” of the clinical notes, 13 (1%) “none of it.” |
| Mishuris, 2015 USA | My HealtheVet (MHV); descriptive, quantitative; 14 patients | Barriers: limited knowledge, satisfaction with current HBPC care, limited computer and Internet access.  Facilitators: desire to learn more about MHV and its potential use; value of surrogates (eg daughter, family member) as intermediaries between Veterans and MHV. |
| Moll, 2018 Sweden | Journalen for patient-accessible HER; descriptive, quantitative; 423,141 patients | 96·58% (2454/2541) of users reported positive experience. Main reason for use was to gain an overview of health status. The portal was important because (1) it makes feel more informed; (2) it improves communication with medical staff; and (3) it results in a better understanding of one’s health status. The most important available resource was test results. |
| Portz, 2019 USA | My Health Manager (Kaiser Permanente Colorado patient portal); qualitative descriptive study; 24 patients | For users the portal was useful in communicating with provider, accessing health information, saving time and money, and addressing health concerns without a clinic visit. Users had logged in on an average 17·1 (SD 28·3) days before recruitment. The majority of participants, regardless of user status, used email and looked up information on the Web. Eemail Message Center was the most popular feature used. Other commonly used features were medications refill, and lab results viewing. Although participants stated that the website was “pretty easy to use,” both portal user and non-user participants (as shown during the focus group) were relatively negative about the UI and UX. Both portal users and non-users expressed issues regarding apprehension of using the technology. |
| Price-Haywood, 2017 USA | MyOchsner patient portals (Epic System), wearable technology, smartphone mobile applications; descriptive, quantitative; 247 patients | Barriers: online security of information, lack of personalization in using technology, lack of resources, desire for skills or technical support to navigate computers and/or the Internet, cumbersome nature of logging into portal accounts, variation in provider availability for online appointment scheduling and response times to medical messages. |
| Quanbeck, 2018 USA | Seva; interventional, other than RCT; 268 patients | Barriers: paying for the service |
| Riippa, 2014 Finland | No specific portal; interventional, other than RCT; 876 patients | Greater positive change in patient activation was identified among patients diagnosed with a severe condition during the intervention than among patients whose last severe diagnosis was made more than 2 years ago. A severe diagnosis may have an independent immediate effect on patient activation |
| Ronda, 2014 Netherland | Digitaal Logboek; descriptive, quantitative; 1390 patients | Most useful features for users: access to the laboratory values with treatment targets, possibility of rereading clinic consultations, and summary of all controls. Non-users suggested that a summary of upcoming consultations and a summary of medication could be the most useful parts. Non-users and regular users perceived as not as useful: access to laboratory values (71·7%, 383/534 vs 92·3%, 372/403), rereading clinic visits (61·3%, 320/522 vs 89·6%, 360/402), e-messaging (52·0%, 262/504 vs 74·6%, 299/401), and uploading results to the glucose diary (45·3%, 229/506 vs 74·0%, 288/400; all P < 0·001). Most non-users (72·4%) stated that the main reason for not requesting a login was that they were unaware of the existence of the portal. Other barriers were disinterest in managing their disease (28·5%, 216/758) and feelings of inadequacy with the use of computers and Internet (11·6%, 88/758). Patients treated by a general practitioner were more frequently non-users compared to patients treated by an internist (78·8%, 666/846 vs 28·3%, 92/325; p < 0·001) and more users than non-users became aware of the portal through their physician (94·9%, 392/413 vs 48·8%, 102/209; P < 0·001). |
| Schultz, 2018 USA | MyNemours (EPIC's MyChart); descriptive, qualitative; 19 caregivers | Advantages of portal use for caregivers: “fast” results, visualize trends, “keep a record,” and not interfering with clinic flow.  Perceived disadvantages: “complicated” or easily misunderstood results, learning results prior to disclosure by the team. |
| Stein, 2018 USA | eCare; RCT; 70 patients | Both intervention and control patients preferred hospitals with online record access (85 and 83%, respectively), access to medical records would increase their trust in doctors (85 and 87%, respectively) and their satisfaction with care (91% for each group). Facilitators: patients who received training in accessing and navigating the portal were significantly more likely to register (48% vs. 11%, P < 0·01). Barriers: personal/time constraints and not thinking about accessing it, rather than technical difficulties. |
| Turvey, 2014 USA | My HealtheVet, Blue Button; descriptive, quantitative; 18,398 participants | The odds of using Blue Button increased 60% for each incremental increase in self-rated computer ability and the odds increased 38% if the veteran had a system for organizing his/her health information. The most interesting features for the patients were accessing their laboratory results (71·4%) and current medication list (57·8%). Satisfaction: for 73·3% it helps understand health history better because it is all in one place, 72·2% it helps them monitor their laboratory results, 67·9% it makes it easier to give others important information about their health. |
| Van der Vaart, 2014 Netherlands | Medisch Spectrum Twente; interventional, other than RCT; 360 patients | Lack of Internet access was the most frequent reason for not using the portal (n = 56). Other reasons were: “I planned to but didn’t have time yet” (n = 30), “I’m not interested” (n = 19), “I tried, but something went wrong” (n = 7), and “I don’t know how to visit the portal” (n = 5). The portal was positively appraised and most login users found their personal information “fairly easy” to “very easy” to understand. |
| Wade-Vuturo, 2013 USA | MyHealthAtVanderbilt patient portal; descriptive, quantitative; 54 adults | Benefit: enhanced patient satisfaction, efficiency and quality of face-to-face visits; access to clinical care outside traditional face-to-face visits.  Barriers: Preconceived beliefs or rules about SM, Participants also expressed doubts about reliability of the portal to facilitate a timely and productive message exchange with their providers. Participants frequently described satisfaction with SM for clinically relevant, administrative purposes such as scheduling medical appointments or requesting prescription reauthorizations. Participants described how SM saves time. In addition, participants were satisfied with having multiple communication options because they could select their preferred method of communication (eg, phone, office visit, SM). |
